# The zinc transporter Slc39a5 controls glucose sensing and insulin secretion in pancreatic β-cells via Sirt1- and Pgc-1α-mediated regulation of Glut2

**DOI:** 10.1007/s13238-018-0580-1

**Published:** 2018-10-15

**Authors:** Xinhui Wang, Hong Gao, Wenhui Wu, Enjun Xie, Yingying Yu, Xuyan He, Jin Li, Wanru Zheng, Xudong Wang, Xizhi Cao, Zhuoxian Meng, Ligong Chen, Junxia Min, Fudi Wang

**Affiliations:** 1 School of Public Health, The First Affiliated Hospital, Institute of Translational Medicine, Zhejiang University School of Medicine, Hangzhou, 310058 China; 2 School of Pharmaceutical Sciences, State Key Laboratory of Membrane Biology, Tsinghua-Peking Center for Life Sciences, School of Life Sciences, Tsinghua University, Beijing, 100084 China; 3 Department of Pathology and Pathophysiology, Key Laboratory of Disease Proteomics of Zhejiang Province, Zhejiang University School of Medicine, Hangzhou, 310058 China

**Keywords:** zinc, zinc transporter, pancreatic islets, β-cells, insulin secretion

## Abstract

Zinc levels are high in pancreatic β-cells, and zinc is involved in the synthesis, processing and secretion of insulin in these cells. However, precisely how cellular zinc homeostasis is regulated in pancreatic β-cells is poorly understood. By screening the expression of 14 *Slc39a* metal importer family member genes, we found that the zinc transporter Slc39a5 is significantly down-regulated in pancreatic β-cells in diabetic *db*/*db* mice, obese *ob*/*ob* mice and high-fat diet-fed mice. Moreover, β-cell-specific *Slc39a5* knockout mice have impaired insulin secretion. In addition, *Slc39a5*-deficient pancreatic islets have reduced glucose tolerance accompanied by reduced expression of Pgc-1α and its downstream target gene Glut2. The down-regulation of Glut2 in *Slc39a5*-deficient islets was rescued using agonists of Sirt1, Pgc-1α and Ppar-γ. At the mechanistic level, we found that Slc39a5*-*mediated zinc influx induces Glut2 expression via Sirt1-mediated Pgc-1α activation. These findings suggest that Slc39a5 may serve as a possible therapeutic target for diabetes-related conditions.

**Electronic supplementary material:**

The online version of this article (10.1007/s13238-018-0580-1) contains supplementary material, which is available to authorized users.

## Introduction

Diabetes is the one of most common chronic diseases in the global population, giving rise to tissue damage and secondary complications. Impaired insulin secretion has become the principal cause of the recent drastic increase in the incidence of diabetes (Ashcroft and Rorsman, 2012). Most recently, the zinc and zinc transporters have been increasing associated with diabetes (Rutter et al., 2016).

Zinc, an essential trace element, has long been recognized as an important player in the biosynthesis, processing and secretion of insulin (Li, 2014). During exocytosis, zinc is co-secreted with insulin into the extracellular space and can be transported back into β-cells and neighboring cells (Franklin et al., 2005). Moreover, perfusing pancreatic islets and MIN6 cells (a pancreatic β-cell line) with stimulating levels of glucose or KCl drastically increases the rate of zinc transport into the cytosol (Gyulkhandanyan et al., 2006). In addition, in diabetes, pancreatic zinc levels are reduced by approximately half compared to levels in the non-diabetic pancreas (Scott and Fisher, 1938). In contrast, rats fed a zinc-deficient diet have reduced serum and total pancreatic zinc levels, but normal zinc levels in pancreatic islets of Langerhans (Sondergaard et al., 2006). Although several studies have investigated zinc levels in pancreatic islets, how zinc homeostasis is regulated under physiological and pathological conditions remains unclear.

In mammals, zinc homeostasis is regulated primarily by two transporter family members, with 14 genes encoding the SLC39A importers (Zrt- and Irt-like proteins, or ZIPs) and 10 genes encoding the SLC30A exporters (zinc efflux transporters, or ZnTs) (Huang and Tepaamorndech, 2013; Jeong and Eide, 2013). Among these zinc transporters, Slc30a8 is the most well-studied zinc exporter in pancreatic β-cells. Slc30a8 is highly expressed in β-cells, where it plays a role in insulin processing and secretion by transporting zinc via granules (Pound et al., 2009). In contrast, the SLC39A family of zinc importers has not been studied in detail. Down-regulation of *Slc39a6* and *Slc39a7* expression significantly decreases: I) cytosolic zinc influx, II) the insulin granule exocytosis rate, and III) insulin secretion in both MIN6 β-cells and primary mouse islet cells (Liu et al., 2015). Conversely, increasing cellular zinc levels by overexpressing Slc39a6 or Slc39a7 has no effect on insulin secretion in healthy β-cells, suggesting that zinc does not directly stimulate insulin secretion, but rather suggesting that intracellular zinc content must be tightly regulated in order to maintain β-cell function (Liu et al., 2015). Of the 14 Slc39a family members, only Slc39a4 has been knocked out in murine β-cells, with no resulting effect on glucose-stimulated insulin secretion (GSIS) *in vivo* (Hardy et al., 2015).

To investigate further the role of Slc39a family members in diabetes, we measured the mRNA levels of all 14 Slc39a family members in pancreatic β-cells in three mouse models of diabetes. Our screen revealed that *Slc39a5* was the only gene with consistently reduced expression. We therefore generated β-cell-specific *Slc39a5* knockout mice and global *Slc39a5* knockout (*Slc39a5*^−/−^) mice in order to examine the function of *Slc39*a5 in β-cells *in vivo*.

## Results

### *Slc39a5* is significantly down-regulated in pancreatic islets obtained from high-fat diet (HFD)-fed, *ob*/*ob* and *db*/*db* mice

**Figure 1:**
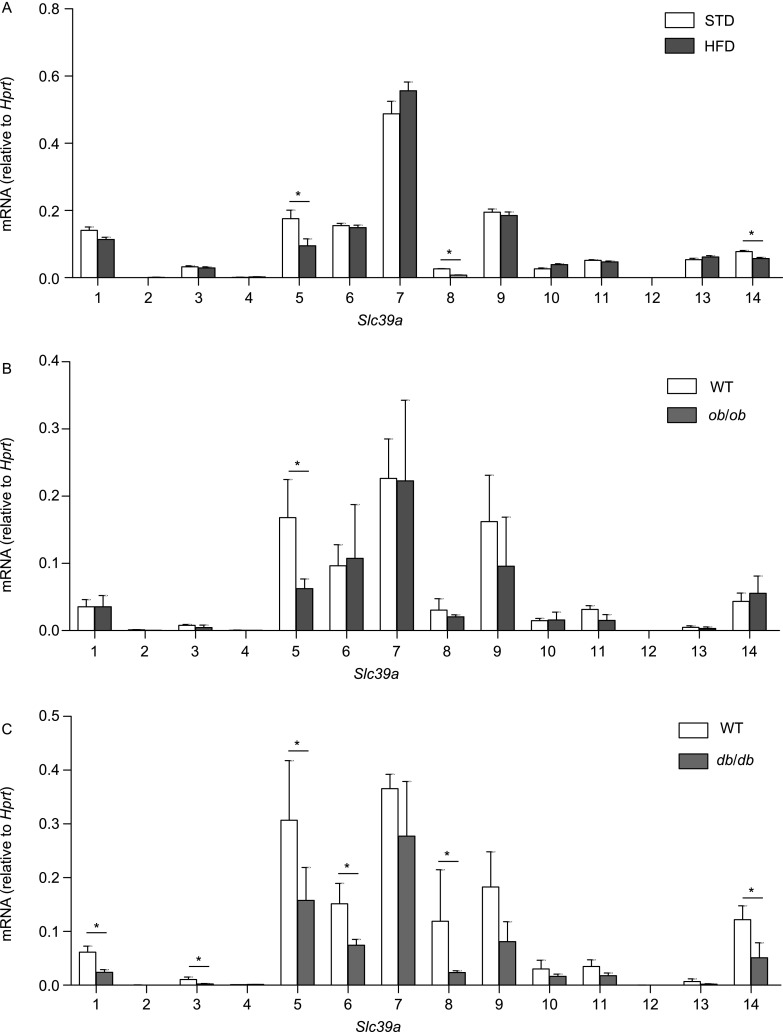
**Summary of mRNA levels of all** ***Slc39a*** **gene family members measured in pancreatic islets isolated from HFD-fed,** ***ob*/*ob*** **and** ***db*/*db*** **mice**. The mRNA levels of all 14 *Slc39a* genes were measured in pancreatic islets isolated from wild-type mice fed either a standard chow diet (STD) or a HFD (A), wild-type and *ob*/*ob* mice (B), and wild-type and *db*/*db* mice (C); *n* = 3 mice per group. *P < 0.05 (Student’s *t*-test)

First, we measured the mRNA levels of all 14 *Slc39a* genes (*Slc39a1* through *Slc39a*14) in the pancreatic islets of HFD-fed, *ob*/*ob*, and *db*/*db* mice. Mice carrying a spontaneous mutation in leptin (*ob*/*ob*) and mice carrying a spontaneous mutation in the leptin receptor (*db*/*db*) were first characterized back in 1950 (Ingalls et al., 1950) and 1966 (Hummel et al., 1966), respectively. Together, these three mouse models have been widely used to investigate the pathological processes that underlie obesity and diabetes(Fellmann et al., 2013). As shown in Figure 1, our analysis revealed that the expression level of *Slc39a5* was decreased by 46% (HFD-fed mice), 63% (*ob*/*ob* mice) and 49% (*db*/*db* mice) in these models relative to control littermates. Among all 14 *Slc39a* genes, only *Slc39a5* was significantly reduced in all three mouse models.

### Impaired glucose tolerance and GSIS in *Slc39a5*-deficient pancreatic β-cells

**Figure 2:**
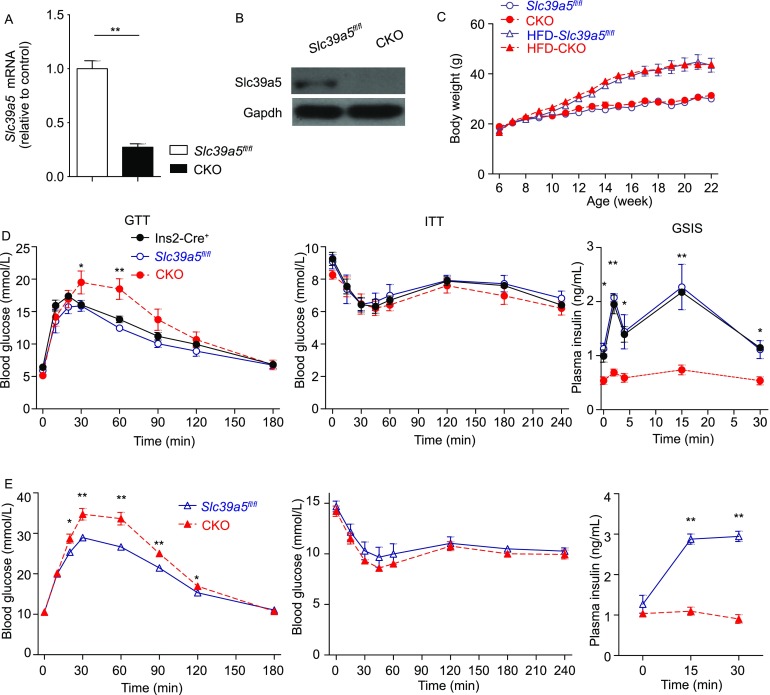
**β-cell**-**specific** ***Slc39a5*** **knockout (CKO) mice have impaired glucose tolerance and glucose-stimulated insulin secretion**. (A and B) *Slc39a5* mRNA (A) and Slc39a5 protein levels (B) were measured in pancreatic islets isolated from *Slc39a5*^*fl/fl*^ and CKO mice (*n* = 3 mice per group). (C) The body weight of *Slc39a5*^*fl*/*fl*^ and CKO mice fed either normal chow diet or a high-fat diet starting at 6 weeks of age (*n* = 6 mice per group). (D and E) Control (*Slc39a5*^*fl*/*fl*^ or Ins2-Cre^+^) and CKO mice fed either normal chow diet (D) or a high-fat diet (E), followed by the glucose tolerance test (GTT, left), insulin tolerance test (ITT, middle), and glucose-stimulated insulin secretion (GSIS, right) (*n* = 6 mice per group). *P < 0.05 and **P < 0.01 (Student’s *t*-test)

Next, to study the function of Slc39a5 *in vivo*, we generated a conditional *Slc39a5* knockout mouse using a LoxP insertion strategy (Fig. S1). Crossing the resulting *Slc39a5*^*fl*/*fl*^ mice with mice that express Cre recombinase driven by the rat insulin (*Ins2*) promoter yielded *Slc39a5*^*fl*/*fl*^;Ins2-Cre^+^ mice (hereafter referred to as CKO mice), which lack Slc39a5 expression selectively in pancreatic β-cells (Fig. S2A). Loss of *Slc39a5* expression in the pancreatic islets of CKO mice was confirmed at the mRNA (Fig. 2A) and protein (Fig. 2B) levels. The expression levels of other *Slc39a* genes were quantified using qPCR in islets of *Slc39a5* knockout and their controls. As shown in Figure S3, we found that there are no compensational changes of other Slc39a genes in *Slc39a5* deficient islets. The offspring of *Slc39a5*^*fl*/*fl*^ × Ins2-Cre^+^ crosses were born at the expected Mendelian ratio.

The body weight of CKO mice was similar to control (*Slc39a5*^*fl*/*fl*^) littermates, even at six months of age (Fig. 2C). In addition, fasting glucose levels were similar between CKO mice and *Slc39a5*^*fl*/*fl*^ mice. However, when challenged with an intraperitoneal (i.p.) injection of glucose (1 g/kg body weight) in a glucose tolerance test (GTT), blood glucose levels in CKO mice were significantly increased both 30 and 60 min after stimulation compared to control (*Slc39a5*^*fl*/*fl*^ or Ins2-Cre^+^) littermates (Fig. 2D, left). In contrast, insulin sensitivity was similar between CKO and control (*Slc39a5*^*fl*/*fl*^ or Ins2-Cre^+^) mice (Fig. 2D, middle). Finally, intraperitoneal GSIS was detected at 0, 2, 5, 15 and 30 min following glucose injection. Both first and second phases of GSIS were significantly increased in *Slc39a5*^*fl*/*fl*^ or Ins2-Cre^+^ mice, but were blunted in CKO mice (Fig. 2D, right).

Next, we examined the role of Slc39a5 in the pathogenesis of HFD-induced diabetes by feeding 6-week-old *Slc39a5*^*fl*/*fl*^ and CKO mice with a high-fat diet containing 60% of calories derived from fat. Although both groups progressively gained more weight compared to their respective control-fed groups, we found no difference in body weight between HFD-*Slc39a5*^*fl*/*fl*^ and HFD-CKO, even after 16 weeks on the HFD (Fig. 2C). When both HFD-*Slc39a5*^*fl*/*fl*^ and HFD-CKO mice were subjected to a GTT assay, the HFD-CKO mice had significantly increased blood glucose levels starting 30 min after glucose injection, and this increase lasted until 120 min after glucose injection (Fig. 2E, left). In contrast, although HFD significantly decreased insulin sensitivity in both the CKO and *Slc39a5*^*fl*/*fl*^ mice, there was no difference between HFD-CKO and HFD-*Slc39a5*^*fl/fl*^ mice (Fig. 2E, middle). Finally, HFD-fed *Slc39a5*^*fl*/*fl*^ mice had a prolonged increase in insulin secretion following glucose injection; in contrast—and similar to CKO mice fed a standard diet—GSIS was unchanged in the HFD-fed CKO mice (Fig. 2E, right). Since the majority mice used in this study were 3 to 6 months old, we next address the possibility of age-effect on the observed phenotypes. GTT has been conducted for 2 or 12 months old mice, and CKO mice showed similar phenotypes among different ages (Fig. S4). Taken together, these results show that β-cell-specific deletion of *Slc39a5* reduces both glucose tolerance and GSIS, but has no effect on insulin sensitivity under either normal or high-fat dietary conditions.

### Pancreatic islet function is impaired in CKO mice

**Figure 3:**
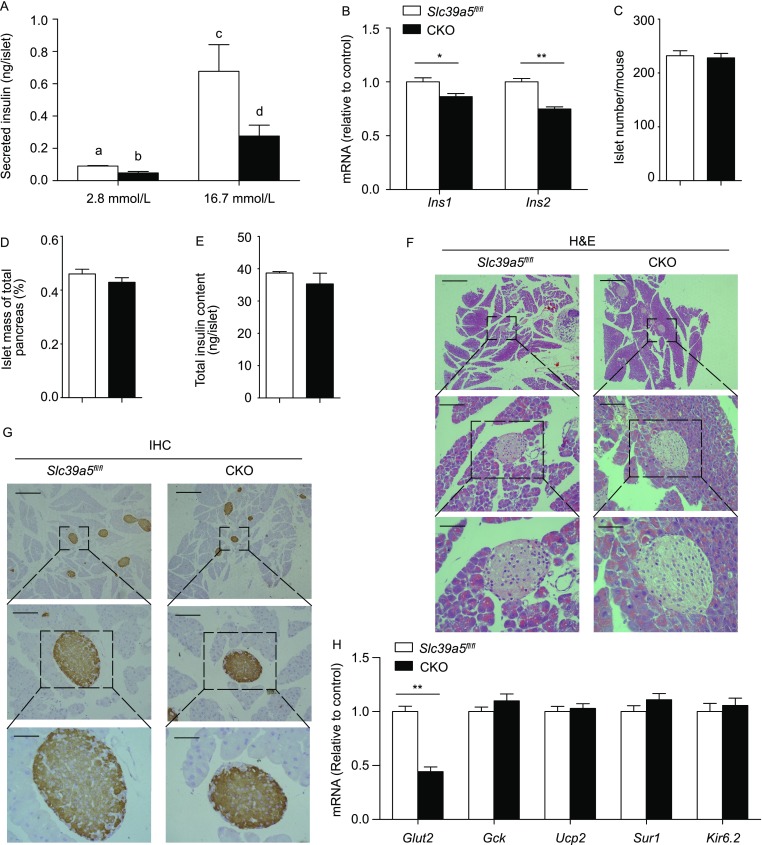

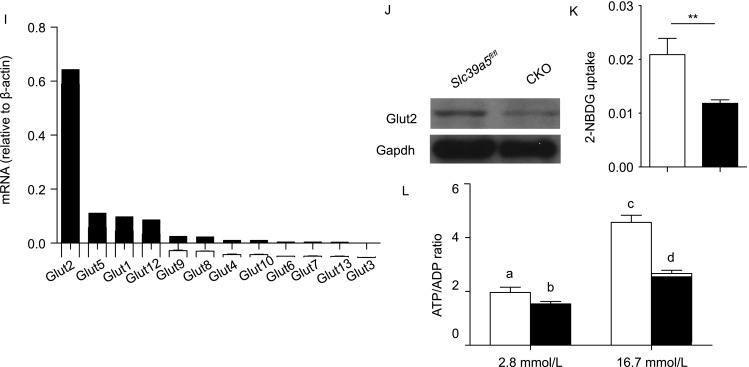
**CKO pancreatic β-cells have impaired function**. (A) Insulin secretion measured in pancreatic islets isolated from *Slc39a5*^*fl*/*fl*^ and CKO mice and incubated in 2.8 or 16.7 mmol/L glucose (*n* = 5 mice per group). (B) *Ins1* and *Ins2* mRNA levels were measured in islets obtained from *Slc39a5*^*fl*/*fl*^ and CKO mice (*n* = 3 mice per group). (C–E) Summary of the number of pancreatic islets (C), relative islet mass (D), and total islet insulin content (E) in *Slc39a5*^*fl*/*fl*^ and CKO mice (*n* = 3 mice per group). (F) Example H&E-stained pancreatic islets in *Slc39a5*^*fl*/*fl*^ and CKO mice. (G) Example immunohistochemical staining for insulin in pancreatic islets from *Slc39a5*^*fl*/*fl*^ and CKO mice. The scale bars in panels (F) and (G) represent 250 μm (top), 50 μm (middle) and 25 μm (bottom). (H) The mRNA levels of the indicated genes were measured in pancreatic islets from *Slc39a5*^*fl*/*fl*^ and CKO mice and are expressed relative to the respective *Slc39a5*^*fl*/*fl*^ level (*n* = 4 mice per group). (I) The mRNA levels of the indicated *Glut* genes were measured in pancreatic islets isolated from wild-type mice and are expressed relative to *β-actin* (*n* = 3 mice per group). (J) Western blot analysis of Glut2 protein in islets from *Slc39a5*^*fl*/*fl*^ and CKO mice. (K) Islets were obtained from *Slc39a5*^*fl*/*fl*^ and CKO mice and incubated with 2-NBDG (20 mmol/L) (*n* = 4 mice per group). (L) Islets were obtained from *Slc39a5*^*fl*/*fl*^ and CKO mice, incubated in 2.8 or 16.7 mmol/L glucose, and the ATP/ADP ratio was measured (*n* = 5 mice per group). The data in panels (A) and (L) were analyzed by ANOVA, and groups with different letters differed significantly; the data in panels (B–E), (H) and (K) were analyzing using a Student’s *t*-test (*P < 0.05 and **P < 0.01)

To examine the functional changes induced by the loss of *Slc39a5* in pancreatic β-cells, primary pancreatic islets were isolated from CKO and *Slc39a5*^*fl*/*fl*^ mice, cultured *ex vivo*, and then stimulated with 2.8 or 16.7 mmol/L glucose. Stimulation with 16.7 mmol/L glucose significantly increased insulin secretion in both *Slc39a5*^*fl*/*fl*^ and CKO islets; however, insulin secretion was significantly reduced in CKO islets compared to *Slc39a5*^*fl*/*fl*^ islets (Fig. 3A). In addition, the mRNA levels of both *Ins1* and *Ins2* were significantly lower in CKO islets compared to control islets (Fig. 3B). On the other hand, the number of islets, the mass of the islet relative to the total pancreas, and the total insulin content were similar between *Slc39a5*^*fl*/*fl*^ and CKO mice (Fig. 3C–E). Moreover, islet morphology (Fig. 3F) and pancreatic insulin staining (Fig. 3G) were also similar between *Slc39a5*^*fl*/*fl*^ and CKO mice. These results suggest that Slc39a5 regulates β-cell function primarily via GSIS, not via islet integrity or insulin storage.

Next, we measured the mRNA levels of several genes that encode key regulators of GSIS, including *Glut2*, *Gck*, *Ucp2*, *Sur1* and *Kir6.2*. We found that *Glut2* was the only gene significantly down-regulated in CKO islets (Fig. 3H). Glut2 is the major glucose transporter expressed in β-cells (Fig. 3I), and consistent with our mRNA analysis, Glut2 protein levels were significantly decreased in CKO islets (Fig. 3J). In addition, using a 2-NBDG uptake assay, we found that CKO islets have impaired glucose uptake compared to control (Fig. 3K). Finally, the ATP/ADP ratio was significantly lower in CKO islets compared to control islets following stimulation with 16.7 mmol/L glucose (Fig. 3L).

### Slc39a5 regulates insulin secretion via Pgc-1α

**Figure 4:**
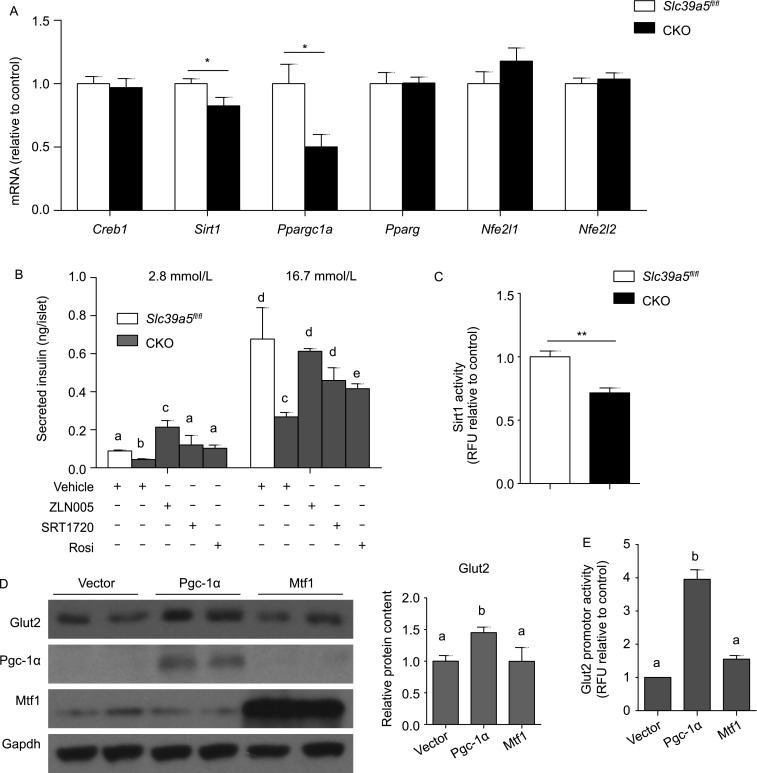
**Slc39a5-regulated insulin secretion in β-cells is mediated by Pgc-1α**. (A) *Creb1*, *Sirt1*, *Ppargc1a*, *Pparg*, *Nfe2l1* and *Nfe2l2* mRNA levels were measured in pancreatic islets from *Slc39a5*^*fl*/*fl*^ and CKO mice (*n* = 4 mice per group). (B) Secreted insulin levels were measured in pancreatic islets isolated from *Slc39a5*^*fl*/*fl*^ and CKO mice after treated with ZLN005, SRT1720, or rosiglitazone (Rosi) and stimulated with 2.8 or 16.7 mmol/L glucose. (C) Sirt1 enzyme activity was measured in islets from *Slc39a5*^*fl*/*fl*^ and CKO mice (*n* = 4 mice per group). (D) MIN6 cells were transfected with either Pgc-1α or Mtf1, and Western blot analysis was performed for the indicated proteins. The relative Glut2 expresion was quantified by using integrated optical densitometry of blots. (E) *Glut2* promoter activity was measured using a dual luciferase reporter assay in MIN6 cells transfected with either Pgc-1α or Mtf1 (*n* = 3 per group). The data in panels (B) and (E) were analyzed by ANOVA, and groups with different letters differed significantly; the data in panels a and c were analyzed using a Student’s *t*-test (*P < 0.05 and **P < 0.01)

In an attempt to identify the underlying mechanism by which Slc39a5 regulates GSIS, we measured the mRNA levels of genes involved in the major signaling pathways related to *Glut2*, *Ins1* and *Ins2* expression (Huang et al., 2010; Wang et al., 2014a; Wang et al., 2014b). Our analysis revealed that genes involved in the antioxidant responsive element (ARE) and metal responsive element (MRE) signaling pathways are down-regulated in CKO islets compared to control islets (Figs. 4A and S5A), whereas genes in the glucocorticoid receptor (GR), nuclear factor of activated T-cells (NFAT) and wingless-type MMTV integration site (WNT) pathways did not differ between CKO and control mice (Fig. S5B–D). Specifically, the mRNA levels of *Sirt1*, *Ppargc1a* and *Mtf1* expression were significantly lower in CKO islets compared to *Slc39a5*^*fl*/*fl*^ islets (Figs. 4A and S5A).

Next, we treated islets with the Pgc-1α activator ZLN005 and found that ZLN005 (20 μmol/L for 12 h) rescued insulin in CKO islets (Fig. 4B). Similarly, both the Sirt1 activator SRT1720 (2 μmol/L for 12 h) and the Ppar-γ agonist rosiglitazone (1 μmol/L for 12 h) rescued insulin secretion in CKO islets (Fig. 4B). Moreover, consistent with our finding of reduced *Sirt1* mRNA in CKO islets, we found significantly reduced Sirt1 activity in CKO β-cells compared to control cells (Fig. 4C). In addition, overexpressing Pgc-1α in MIN6 cells increased Glut2 expression, whereas overexpressing Mtf1 had no effect on Glut2 expression, even though Mtf1 expression was decreased in CKO islets (Fig. 4D). Finally, overexpressing Pgc-1α, but not Mtf1, significantly up-regulated *Glut2* promotor activity in MIN6 cells (Fig. 4E). Taken together, these results indicated that loss of *Slc39a5* expression in mouse pancreatic islets significantly reduces the expression and activity of Sirt1, as well as *Ppargc1a* expression, and activating ARE signaling rescues both Glut2 expression and impaired insulin secretion.

Given the role that Pgc-1α plays in the mitochondria, we looked for morphological changes in the mitochondria of CKO and control β-cells using transmission electron microscopy. We found that CKO β-cells—but not control β-cells—have mitochondrial swelling (Fig. S6A). In addition, Rhodamine 123 uptake was significantly higher in CKO cells compared to control cells (Fig. S6B), suggesting altered mitochondrial membrane potential. In contrast, the expression of mitochondrial complexes I, II, III, IV and V in pancreatic islets did not differ between CKO and control mice (Fig. S6C). Similarly, we found no difference between CKO and control islets with respect to expression of the mitochondrial genes *Atp6*, *Nd4*, *Cytb* or *Cox3* (Fig. S6D), or the electron transfer chain complex genes *Ndufs3*, *Cycs*, *Cox8b*, *Atp5c1*, *Atp5d* or *Atp5* (Fig. S6E). Thus, we conclude that deleting Slc39a5 expression in pancreatic β-cells only mildly affects mitochondrial function.

### Zinc-dependent effects of Slc39a5 deletion on insulin secretion

**Figure 5:**
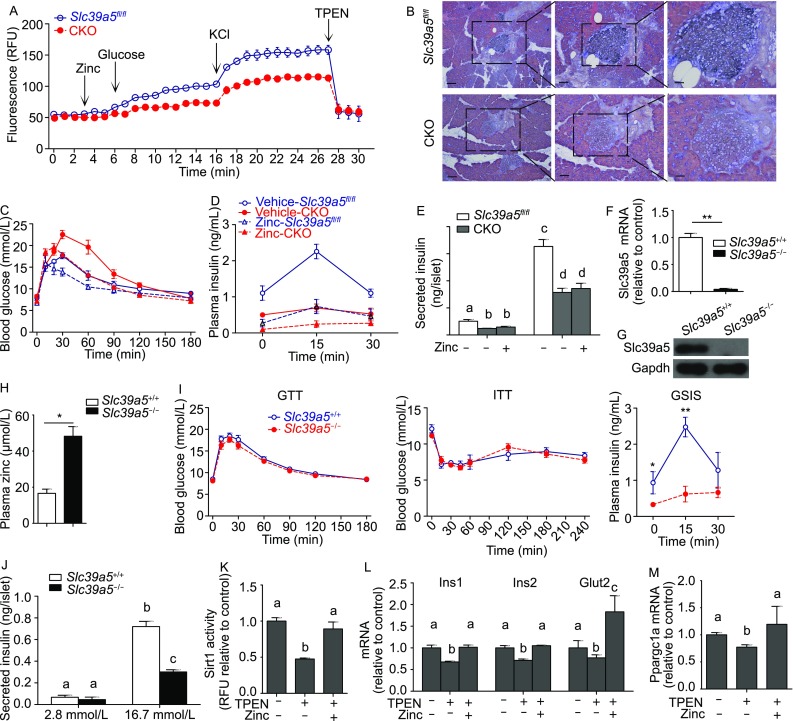
***Slc39a5*** **deficiency reduces zinc,** ***Ppargc1a*** **expression and insulin secretion**. (A) Time course of zinc content measured in pancreatic islets isolated from *Slc39a5*^*fl*/*fl*^ and CKO mice and loaded with the fluorescent zinc indicator FluoZin-3AM. Where indicated, ZnCl_2_ (10 μmol/L), glucose (16.7 mmol/L), KCl (30 mmol/L), and TPEN (10 μmol/L) were applied (*n* = 5 mice per group). (B) Timm’s zinc staining of pancreas islets from *Slc39a5*^*fl*/*fl*^ and CKO mice. From left to right, the scale bars indicate 100 μm, 50 μm and 25 μm. GTT (C) and GSIS (D) were measured in *Slc39a5*^*fl*/*fl*^ and CKO mice treated with vehicle or ZnSO_4_ (10 mg/kg body weight, i.p.) for 5 consecutive days (*n* = 6 mice per group). (E) Secreted insulin levels were measured in pancreatic islets isolated from *Slc39a5*^*fl*/*fl*^ and CKO mice after treated with zinc and stimulated with 2.8 or 16.7 mmol/L glucose. (F) *Slc39a5* mRNA was measured in pancreatic islets isolated from *Slc39a5*^−*l*−^ and *Slc39a5*^*+l+*^ mice (*n* = 3 mice per group). (G) Western blot analysis of Slc39a5 protein in isolated islets from *Slc39a5*^−*l*−^ and *Slc39a5*^*+l+*^ mice; the data are representative of 3 mice each. (H) Plasma zinc content was measure in *Slc39a5*^−*l*−^ and *Slc39a5*^*+l+*^ mice (*n* = 3 mice per group). (I) GTT, ITT, and GSIS were performed in *Slc39a5*^−*l*−^ and *Slc39a5*^*+l+*^ mice (*n* = 6 mice each). (J) Secreted insulin levels were measured in pancreatic islets isolated from *Slc39a5*^−*l*−^ and *Slc39a5*^*+l+*^ mice after stimulated with 2.8 or 16.7 mmol/L glucose (*n* = 5 mice per group). (K) Sirt1 activity was measured in islets isolated from wild-type mice treated with TPEN or TPEN plus zinc (*n* = 3 mice per group). (L) mRNA levels of *Ins1, Ins2* and *Glut2* were measured in islets from wild-type mice treated with TPEN or TPEN plus zinc (*n* = 4). (M) mRNA levels of *Ppargc1a* were measured in the islets of wild-type mice treated with TPEN or TPEN plus zinc (*n* = 4 mice each). The data in panels (E) and (J–M) were analyzed by ANOVA, and groups with different letters differed significantly; the data in panels (A), (C), (D), (F), (H) and (I) were analyzed using a Student’s *t*-test (*P < 0.05 and **P < 0.01)

Next, we examined the capacity of CKO β-cells to take up zinc by measuring zinc levels in CKO and control β-cells following treatment with glucose (16.7 mmol/L) and KCl (30 mmol/L) in the continued presence of 10 μmol/L ZnCl_2_. We found that both glucose- and KCl-induced zinc uptake was significantly impaired in CKO β-cells compared to control cells (Fig. 5A). In addition, Timm’s staining of pancreatic islets to stain zinc showed significantly reduced zinc in CKO mice compared to control mice (Fig. 5B).

We then treated CKO mice with ZnSO_4_ (10 mg/kg/day, i.p.) for 5 consecutive days and found that this treatment restored glucose tolerance to the same level as untreated *Slc39a5*^*fl*/*fl*^ mice (Fig. 5C). In contrast, this treatment had no significant effect on GSIS in CKO mice (Fig. 5D). Furthermore, zinc rescue experiments in cultured CKO islets were conducted, and the results showed that supplementation of zinc (20 μmol/L for 12 h) in the medium failed to rescue the impaired GSIS in CKO islets (Fig. 5E).

Previous studies showed that global *Slc39a5* knockout (*Slc39a5*^−*l*−^) mice have increased zinc accumulation in the body (Geiser et al., 2013); we generated a global *Slc39a5* knockout (*Slc39a5*^−*l*−^) mouse by crossing our *Slc39a5*^*fl*/*fl*^ mice with CMV-Cre^+^ mice (Fig. S2B). We confirmed the loss of Slc39a5 expression at both the mRNA (Fig. 5F) and protein (Fig. 5G) levels. Compared to *Slc39a5*^*+l+*^ mice, *Slc39a5*^−*l*−^ mice had significantly increased plasma zinc levels (Fig. 5H). We then performed the GTT and ITT in these mice and found no difference between *Slc39a5*^−*l*−^ and *Slc39a5*^*+l+*^ mice (Fig. 5I, left and middle). However, GSIS was significantly lower in the *Slc39a5*^−*l*−^ mice compared to *Slc39a5*^*+l+*^ littermates (Fig. 5I, right). We then measured *ex vivo* GSIS in primary pancreatic islets isolated from *Slc39a5*^−*l*−^ and *Slc39a5*^*+l+*^ mice following stimulation with 2.8 or 16.7 mmol/L glucose. Consistent with our *in vivo* results, *Slc39a5*^−*l*−^ islets had significantly reduced GSIS compared to *Slc39a5*^*+l+*^ islets (Fig. 5J).

Next, we treated islets obtained from wild-type mice treated with TPEN (N,N,N’,N’-Tetrakis (2-pyridylmethyl) ethylenediamine) in the absence or presence of zinc. We found that TPEN reduced Sirt1 activity in wild-type islets, and zinc treatment restored Sirt1 activity to baseline levels (Fig. 5K). Moreover, TPEN treatment reduced the levels of *Ins1, Ins2*, *Glut2* and *Ppargc1a* mRNA, and zinc treatment restored the full expression of all four genes (Fig. 5L and 5M). Taken together, these results suggest that *Slc39a5* deficiency impairs zinc uptake, ARE-mediated signaling, and GSIS in pancreatic β-cells.

## Discussion

Genome-wide association studies found that a nonsynonymous polymorphism in the human *SLC30A8* gene is associated with type 2 diabetes (T2D) (Saxena et al., 2007; Sladek et al., 2007). In mice, deleting *Slc30a8* expression selectively in pancreatic β-cells causes reduced glucose tolerance accompanied by impaired insulin synthesis, processing, and secretion (Wijesekara et al., 2010). Recently, Li et al. reported that transgenic mice that overexpress human *SLC30A8* with the R325W polymorphism have reduced levels of zinc and proinsulin in pancreatic islets (Li et al., 2017). After consuming a high-fat diet, however, these mice have increased glucose tolerance (Li et al., 2017), supporting the notion that zinc plays an important role in maintaining β-cell function in pancreatic islets. In this study, since only *Slc39a5* was consistently down-regulated in all three obese T2D mouse models, we mainly focused on functional characterization of the role of *Slc39a5* in pancreatic β-cells. However, it will be interesting to see whether other *Slc39a* family members such as Slc39a6 (Liu et al., 2015), Slc39a8 and Slc39a14 also play roles in regulating zinc homeostasis in β-cells.

Despite these findings, the effects of zinc on GSIS remain poorly understood. Previous studies found that robust zinc uptake is related to glucose stimulation and/or high KCl stimulation (Gyulkhandanyan et al., 2006). However, the physiological role and underlying regulatory mechanisms remain unclear. Here, we screened 14 genes that encode Slc39a family members and identified *Slc39a5* as the sole gene down-regulated in three mouse models of obesity. Although the leptin signaling is activated in HFD-induced obese mice (Lin et al., 2000), it is actually absent in *ob*/*ob* and *db*/*db* mice. The expression of *Slc39a5* was significantly reduced in all three obese and diabetes murine models, which suggests that the leptin signaling does not directly regulate *Slc39a5* expression. Using β-cell-specific *Slc39a5* knockout (CKO) mice, we found that: I) these mice have reduced glucose tolerance and impaired GSIS, but no change in fasting glucose levels or insulin sensitivity; II) primary islets isolated from these mice have impaired GSIS, decreased glucose uptake capacity, and reduced Glut2 expression, with no change in islet integrity or insulin storage; and III) CKO islets have decreased zinc influx, Sirt1 activity, and Pgc-1α expression.

Deleting *Slc39a5* selectively in pancreatic β-cells decreased zinc levels in pancreatic islets but did not affect serum zinc levels. Interestingly, global *Slc39a5* knockout mice also have decreased zinc levels in islets as well as increased serum zinc levels. Based on these observations, our β-cell-specific *Slc39a5* knockout mice—and other β-cell-specific *Slc39a* knockout mice—will likely serve as a valuable tool for precisely controlling zinc transport selectively in pancreatic islets and will likely facilitate the study of their physiological function and underlying regulatory mechanisms.

Because zinc has pleiotropic effects on glucose homeostasis (Chimienti, 2013), we tested the effect of zinc on glucose homeostasis in mice. Surprisingly, we found that injecting CKO mice with zinc did not restore GSIS, although it did return GTT to control levels. A previous study reported that zinc has an insulin-like effect on facilitating glucose uptake in periphery tissues mainly through phosphoinositol-3-kinase/AKT signaling and GLUT4 translocation (Tang and Shay, 2001). This insulin-like effect of zinc could be the major factor attributed to the different impacts of zinc treatment on GTT (Fig. 5C) and GSIS (Fig. 5D) in the CKO mice. Slc39a5 also plays a role in zinc homeostasis in the intestine, liver and exocrine pancreatic acinar tissue (Wang et al., 2004), and global deletion of *Slc39a5* causes zinc accumulation in the serum (Geiser et al., 2013), which is consistent with our results. Similar to zinc-treated CKO mice, *Slc39a5*^−/−^ mice also have impaired GSIS compared to control littermates, although they still have normal blood glucose levels. The normal GTT in the *Slc39a5*^−/−^ mice (Fig. 5I) could also result from the increased zinc levels in the circulation (Fig. 5H), which could directly facilitate glucose uptake in periphery tissues despite impaired GSIS (Fig. 5I and 5J). Thus, our results further support the notion that excess zinc reduces blood glucose levels directly via an insulino-mimetic effect, not by stimulating insulin secretion.

One of our significant findings is that Sirt1-Pgc-1α signaling—and its downstream target Glut2—is decreased in pancreatic islets in CKO mice. Mitochondrial morphology and mitochondrial membrane potential were also affected—albeit mildly—in CKO islets. Together, impaired glucose uptake and reduced mitochondrial function result in reduced ATP production and impaired GSIS. In humans, SIRT1 has been associated with T2D (Cruz et al., 2010; Dong et al., 2011). In addition, variants in the *PPARGC1A* gene have been associated with T2D (Bhat et al., 2007; Yang et al., 2011; Chand et al., 2016). Interestingly, the *PPARGC1A* Gly482Ser polymorphism is associated with reduced *PPARGC1A* expression and reduced insulin secretion (Ling et al., 2008). In some T2D patients, the expression of *PPARGC1A* is reduced by 90% and is correlated with impaired insulin secretion in pancreatic islets. Furthermore, using siRNA to knock down *PPARGC1A* in human pancreatic islets reduced insulin secretion by 41% (Ling et al., 2008). Furthermore, we previously reported increased GSIS and increased Glut2 expression in mice treated with the glutathione peroxidase mimic ebselen; these effects were mediated primarily through Pgc-1α-mediated activation of ARE signaling (Wang et al., 2014b). Taken together, the results of the present study provide key insight into the molecular mechanisms underlying impaired islet function in response to β-cell-specific zinc deficiency.

Notably, our data suggest that the phenotypic change of GTT and GSIS was mainly due to impaired insulin secretion but not synthesis. Although the transcripts of *Ins1* and *Ins2* were indeed decreased in the *Slc39a5*-deficient islets, the protein levels of pancreatic insulin measured by total insulin content (Fig. 3E) and immunohistochemistry staining (Fig. 3G) remain unchanged between *Slc39a5*-deficient and control islets. The unchanged protein level of insulin could result from blunted insulin secretion despite the transcripts of *Ins1* and *Ins2* were slightly decreased. In addition, the first and second phases of GSIS were detected and the result clearly showed diminished insulin secretion in both phases (Fig. 2D), which may also implicate a direct impairment of glucose response.

**Figure 6:**
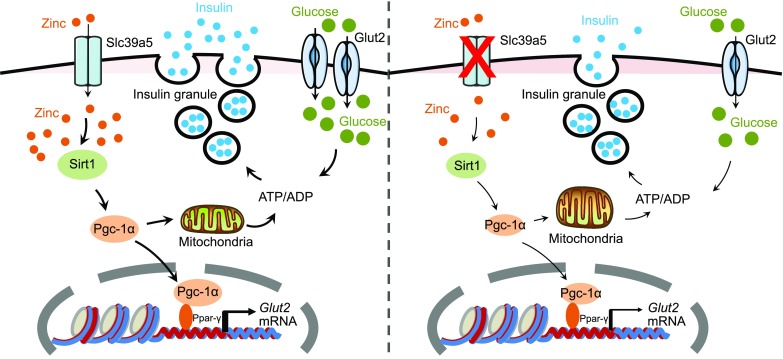
**Schematic model depicting the proposed role of Slc39a5 in pancreatic β-cells**. An appropriate level of intracellular zinc is required for glucose-stimulated ATP synthesis and insulin secretion (left). *Slc39a5* deficiency (right) results in impaired glucose-stimulated zinc uptake, Sirt1-Pgc-1α signaling, Glut2 expression, ATP synthesis and glucose-stimulated insulin secretion

In summary, we functionally characterized the role of the zinc transporter Slc39a5 in pancreatic β-cells. With respect to the underlying mechanism, we show that Slc39a5 regulates GSIS in islets primarily through the zinc-activated Sirt1-Pgc-1α axis (Fig. 6). Our results suggest that Slc39a5 deficiency leads to significantly lower intracellular zinc levels. As a result, zinc deficiency down-regulates Sirt1 activity and Pgc-1α expression, which further affects Glut2 expression and mitochondrial function. Consequently, Slc39a5 deficiency in β-cells attenuates both glucose sensing and insulin secretion. These findings pave the way to better understanding how islet function is regulated by zinc and zinc transporters, and they provide possible therapeutic targets for diabetes. We propose that specific zinc transporters have evolved in order to tightly regulate intracellular zinc homeostasis in pancreatic β-cells under a wide range of metabolic conditions, thereby controlling insulin secretion. Further studies should be designed to dissect the unique functions played by various zinc transporters with respect to regulating β-cell function.

## Materials and methods

### Generation of *Slc39a5*^*fl*/*fl*^ mice, breeding and genotyping

*Slc39a5*-floxed (*Slc39a5*^*fl*/*fl*^) mice containing LoxP sites flanking exon 4 in the *Slc39a5* gene (Shanghai Biomodel Organism Science & Technology Development Co. Ltd) were crossed with Ins2-Cre (Postic et al., 1999) and CMV-Cre(Schwenk et al., 1995) mice to generate β-cell-specific *Slc39a5* knockout (*Slc39a5*^*fl*/*fl*^;Ins2-Cre^+^) and global *Slc39a5* knockout (*Slc39a5*^−/−^) mice, respectively. All mice were kept on the C57BL/6 background. Detailed breeding strategies for generating conditional and global knockout mice were shown in Fig. S2. Tail biopsies were analyzed by genomic polymerase chain reaction (forward primer, CCTCTCACACCAACTGCTCA; reverse primer, AGCTTTGTCTGCCACCTTGT), and 3 different sizes of PCR products, including 735 bp, 959 bp and 528 bp represent wild-type, floxed and knockout alleles for *Slc39a5* gene, respectively. A 350 bp PCR product was generated for *Cre* gene (forward, ATTTGCCTGCATTACCGGTC; reverse, ATCAACGTTTTCTTTTCGG).

### Animal studies and high-fat diet

All animal studies were approved by the Institutional Animal Care and Use Committee at Zhejiang University and were conducted in accordance with National Institutes of Health guidelines for animal care. Leptin mutant (*ob*/*ob*, Stock No: 000632) and leptin receptor mutant (*db*/*db*, Stock No: 000697) mice were obtained from The Jackson Laboratory (Bar Harbor, ME). Most of the experimental mice were 3–6 months of age (with an exception in Fig. S4, 2 and 12 months old mice were applied for GTT) and were housed in plastic cages at a constant ambient temperature under a 12 h/12 h light-dark cycle. The mice were supplied with distilled water and pelleted AIN-76A (Research Diets, New Brunswick, NJ) chow containing standard fat content (12% kcal derived from fat). High-fat diet used in this study was purchased from Research Diets (cat# D12492) with 60% kcal derived from fat (2,205 kcal from lard per 4,057 kcal in diet).

### Reagents

HEPES sodium salt, HEPES, potassium chloride, calcium chloride dihydrate, D-(+)-glucose, collagenase V, insulin, rosiglitazone, ZnCl_2_, ZnSO_4_ and TPEN (an intracellular membrane-permeable chelator for various ions, including zinc) were purchased from Sigma (Saint Louis, MO). Total OXPHOS Rodent WB was purchased from Abcam (Cambridge, MA). The Dual Luciferase Assay Kit was obtained from Promega (Madison, WI). Fluo-4 AM, cell-permeant Rhodamine 123 and 2-NBDG were purchased from ThermoFisher Scientific (Waltham, MA). SRT1720 and ZLN005 were purchased from Selleck (Houston, TX). The zinc detection kit was purchased from Nanjing Jiancheng Bioengineering Institute (Nanjing, JS, China).

### Metabolic assays

The GTT, GSIS and insulin tolerance test (ITT) were conducted as described previously (Wang et al., 2011). For GTT and GSIS, the mice were fasted for 8 h and then injected i.p. with 1 g/kg body weight of glucose. For ITT, the mice were fasted for 2 h and then injected i.p. with 0.5 units/kg body weight of insulin. For blood glucose measurements, blood was drawn from the tail vein at the indicated times, and blood glucose was measured using an Accu-Check glucometer (Roche, Indianapolis, IN). To measure plasma insulin, blood was collected from the retro-orbital plexus; the plasma was isolated, and plasma insulin was measured using a rat/mouse insulin enzyme-linked immunosorbent assay kit with mouse insulin as the standard (Crystal Chem, Downers Grove, IL).

### Pancreatic islet isolation and dissociation

Pancreatic Langerhans’ islets were isolated from individual (fasted overnight for 8 h) mice for different treatments and GSIS tests. Isolation and culture islets were performed as previously described (Wang et al., 2011). Briefly, Langerhans’ islets were isolated from the mice using a standard procedure with minor modifications (Gotoh et al., 1985; Hansen et al., 2012). Pancreas was inflated with 2 mg/mL collagenase V (Sigma) solution, excised, and incubated for 5 min at 37 °C in a water bath for full digestion. Islets were then hand-picked to remove all exocrine tissues. Isolated islets were recovered in RPMI 1640 (Gibco, Grand Island, NY) with 5.5 mmol/L glucose and 10% fetal bovine serum for 2 h before designated treatments. To obtain dissociated cells, pancreatic islets were incubated in 0.125% dispase II (Roche Diagnostics) for 5 min at 37 °C with occasional gentle mixing. The cells were then plated on glass coverslips precoated with poly-L-lysine solution (Sigma) and used for culture, staining and/or treatment.

### Measurements of mRNA and protein levels

Islet mRNA and protein levels were analyzed as described previously (Wang et al., 2014b). Total RNA was extracted from islets using Trizol reagent (Invitrogen, Carlsbad, CA). Reverse transcription was performed using Super Script III reverse transcriptase, RNase-OUT Ribonuclease Inhibitor, and Oligo(dT)12–18 (Invitrogen). Relative mRNA levels were determined using the LightCycler 480 II system (Roche) with the primers listed in Table S1. Islet samples used for Western blot analysis were homogenized in phosphate buffer (50 mmol/L, pH 7.4) containing 0.1% Triton X-100 and protease inhibitor cocktail. A total of 10 μg of protein per lane was used for Western blot analysis. The membranes were first incubated with the respective primary antibodies (rabbit anti-Glut2, anti-Pdx1, or anti-Ucp2; Millipore, Billerica, MA), followed by an anti-rabbit secondary antibody (Bio-Rad, Hercules, CA). Gapdh was used as a loading control (Cell Signaling, Beverly, MA).

### Insulin content and islet morphology

Insulin content, islets number and β-cell mass were determined as described previously (Wang et al., 2008). Briefly, total pancreatic insulin concentration was determined (*n* = 6 mice per genotype) using the insulin enzyme-linked immunosorbent assay kit (Crystal Chem) after acid-ethanol extraction. Islets number was counted (*n* = 9 mice per genotype) using microscope for hand-picked islets from each mouse after full digestion with collagenase V as described above. For immunohistochemistry assay, tissues were fixed in 4% PFA for 30 min at 4 °C, embedded in paraffin, and 8-mm sections were cut, affixed to glass slides, and stained with antibodies. Pancreatic β-cell mass was determined by immunostaining of paraffin-embedded pancreatic sections (*n* = 3 mice × 3 slides per genotype) with a rabbit anti-insulin primary antibody (Abcam), and was quantified using ImageJ software (NIH, Bethesda, ML).

### 2-NBDG uptake test

Dissociated pancreatic islet cells were incubated for 30 min in 20 mmol/L 2-NBDG (ThermoFisher Scientific) in Krebs-Henseleit buffer and measured using a microplate spectrophotometer (Arya et al., 2012).

### ATP/ADP ratio

The ATP/ADP ratio was measured using an ATP/ADP ratio assay kit (Abcam) in accordance with the manufacturer’s instructions (Gray et al., 2011).

### Luciferase assay

The luciferase assay was performed according to the manufacturer’s instructions using the Dual-Luciferase Reporter Assay System (Promega). Luciferase activity was measured using a luminometer (Molecular Devices, San Jose, CA).

### Transmission electron microscopy

Transmission electron microscopy was performed using a Tecnai 10 microscope (FEI, Hillsboro, OR) at the Electron Microscopy Core Facility, Zhejiang University.

### Statistical analysis

Except where indicated otherwise, summary data are presented as the mean ± SEM. The Student’s *t*-test was used to compare two groups. Multiple groups were compared using a one-way ANOVA with Tukey’s *post hoc* test. Differences with a *P*-value < 0.05 were considered statistically significant.

## Electronic supplementary material

Below is the link to the electronic supplementary material.

Supplementary material 1 (DOCX 1203 kb)
