# Supplementary material for: The zinc transporter Slc39a5 controls glucose sensing and insulin secretion in pancreatic β-cells via Sirt1- and Pgc-1α-mediated regulation of Glut2
Source: Protein Cell. 2018 Oct 15;10(6):436–49. doi: 10.1007/s13238-018-0580-1 (PMC6538592; doi:10.1007/s13238-018-0580-1)
Supplement: Supplementary file 1 — Supplementary material 1 (DOCX 1203 kb) [file 13238_2018_580_MOESM1_ESM.docx]

**Supplementary files**

**The zinc transporter Slc39a5 controls glucose sensing and insulin secretion in pancreatic β-cells via Sirt1- and Pgc-1α-mediated regulation of Glut2**

Xinhui Wang, *et al*

**
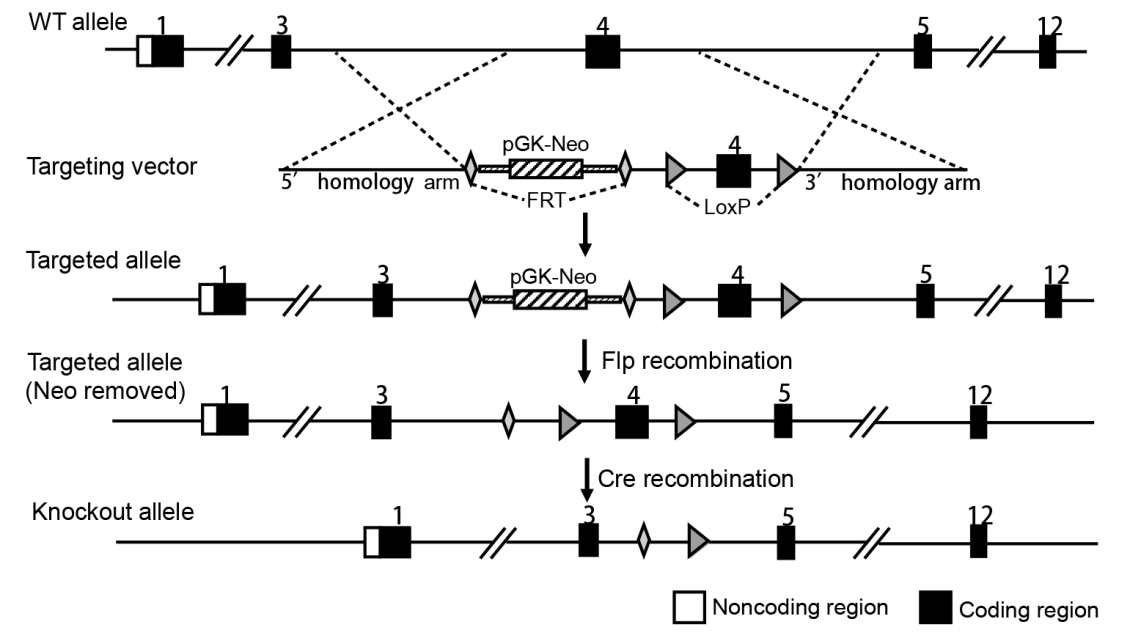
**

**Supplementary Figure 1.** **Targeting strategy for generating *Slc39a5^fl/fl^* and CKO mice.** The targeting vector was designed to excise exon 4 of *Slc39a5* by inserting a LoxP sequence and *Neo* cassette into the region between exons 3 and 5.


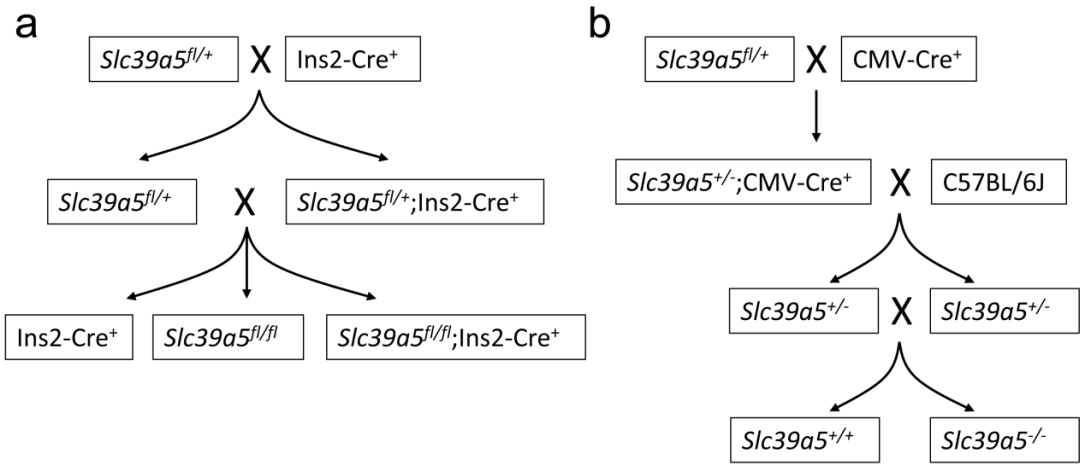


**Supplementary Figure 2.** **Breeding strategies for generating *Slc39a5* conditional and global knockout mice.** Panreatic β-cell conditional (a) and global (b) knockout mice were generated by crossing the *Slc39a5^fl/fl^* mice with Ins2-Cre^+^ or CMV-Cre^+^ mice, respectively.


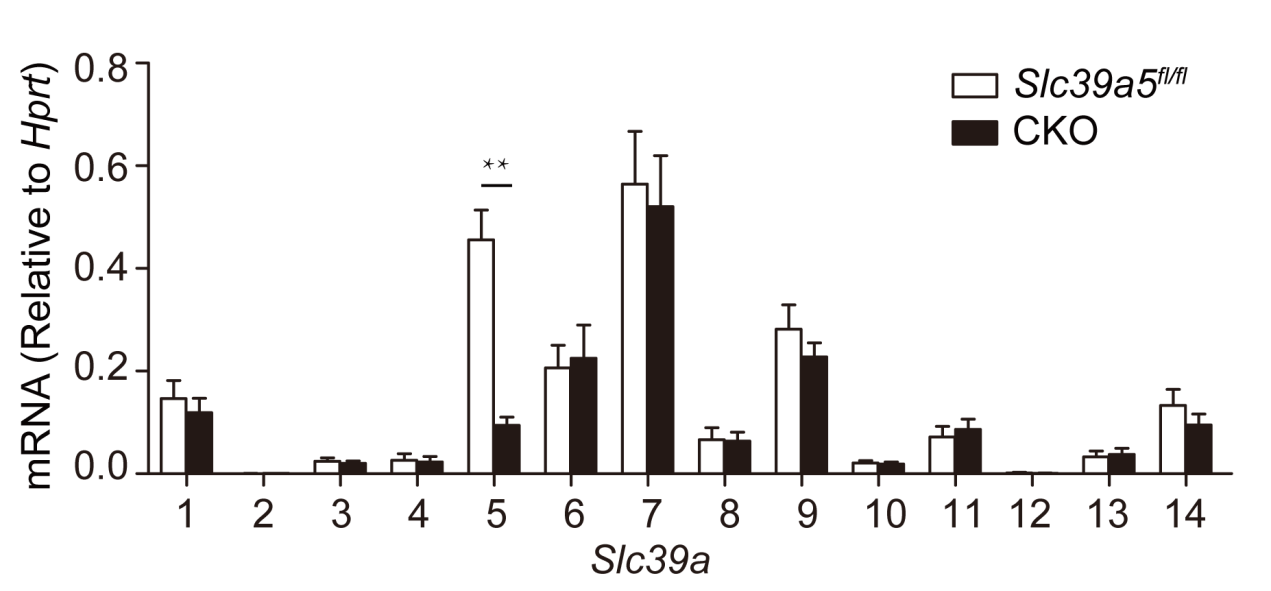


**Supplementary Figure 3.** **Summary of mRNA levels of all *Slc39a* gene family members measured in pancreatic islets isolated from *Slc39a5^fl/fl^* and CKO.** The mRNA levels of all 14 *Slc39a* genes were measured in isolated pancreatic islets (n=8 mice per group). ***p* < 0.01 (Student’s *t*-test).


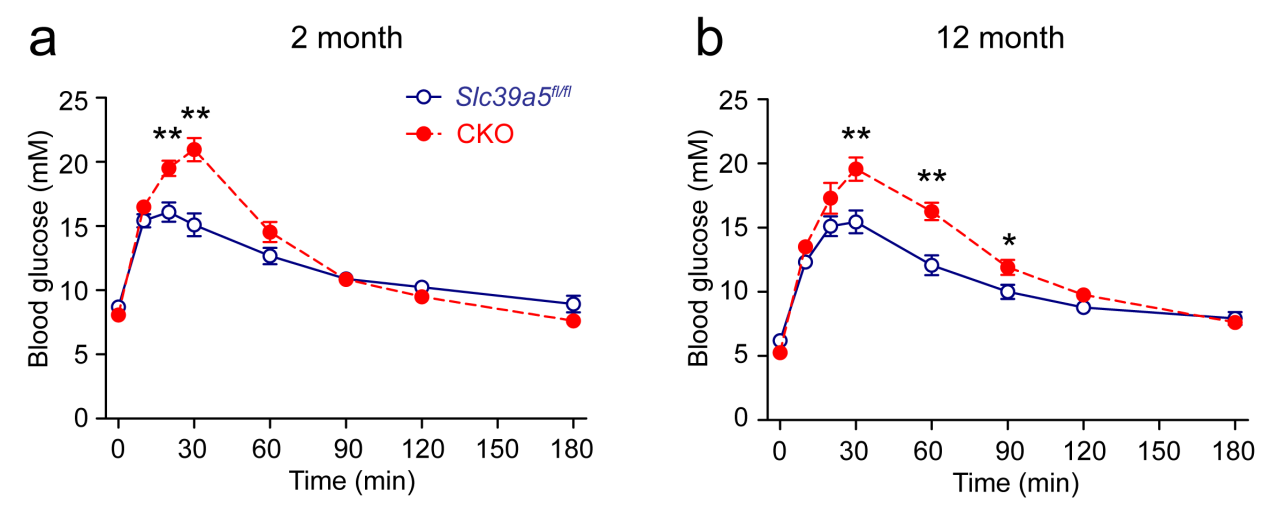


**Supplementary Figure 4. β-cell‒specific *Slc39a5* knockout mice have similar impairments of glucose tolerance in different age.** Glucose tolerance tests were conducted for 2 (a) and 12 (b) month old *Slc39a5^fl/fl^* and CKO mice (n=6 mice per group). **p* < 0.05 and ***p* < 0.01 (Student’s *t*-test).


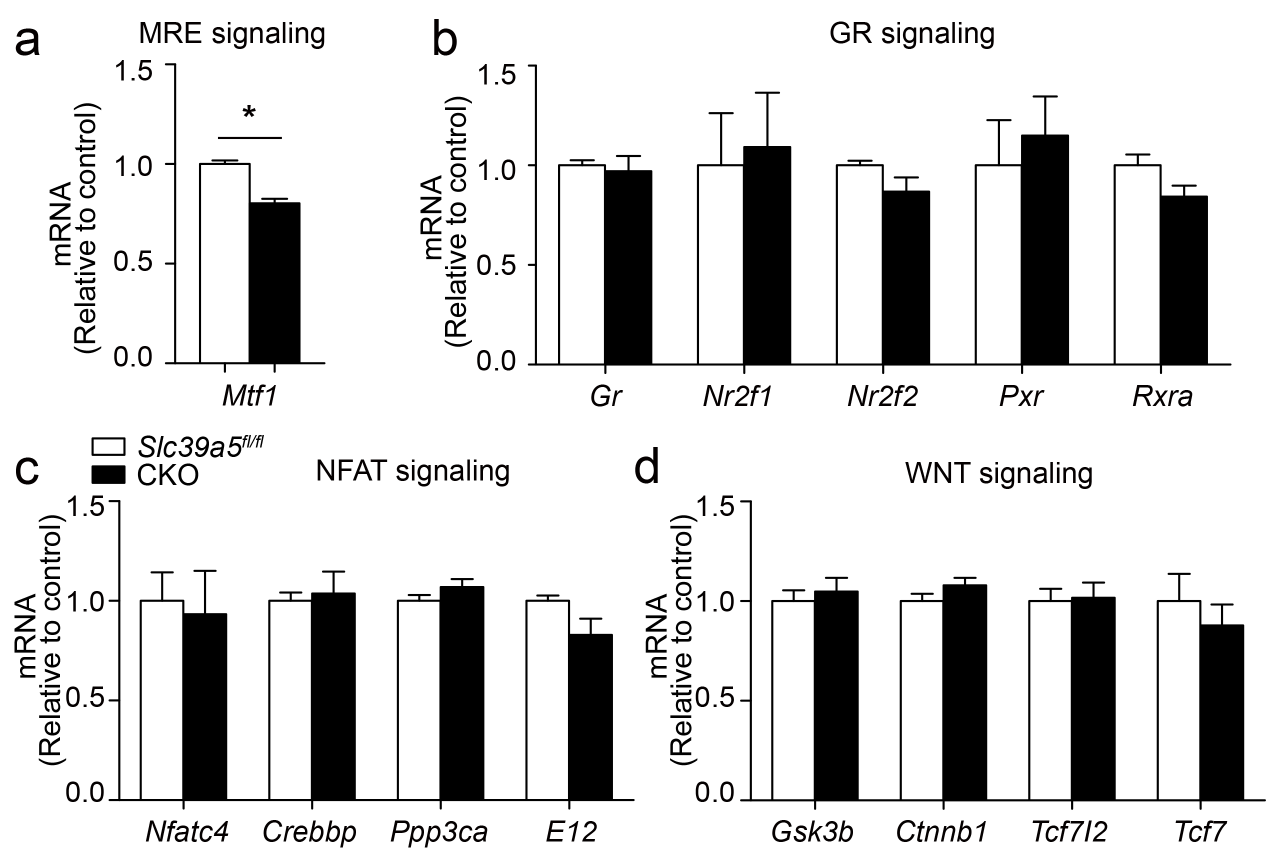


**Supplementary Figure 5.** **The indicated mRNA levels were measured in pancreatic islets isolated from *Slc39a5^fl/fl^* and CKO.** The mRNA levels of the indicated genes in the (a) metal-responsive element (MRE), (b) glucocorticoid receptor (GR), (c) nuclear factor of activated T-cells (NFAT), and (d) wingless-type MMTV integration site (WNT) pathways are shown (n=6 mice per group). **p* < 0.05 (Student’s *t*-test).


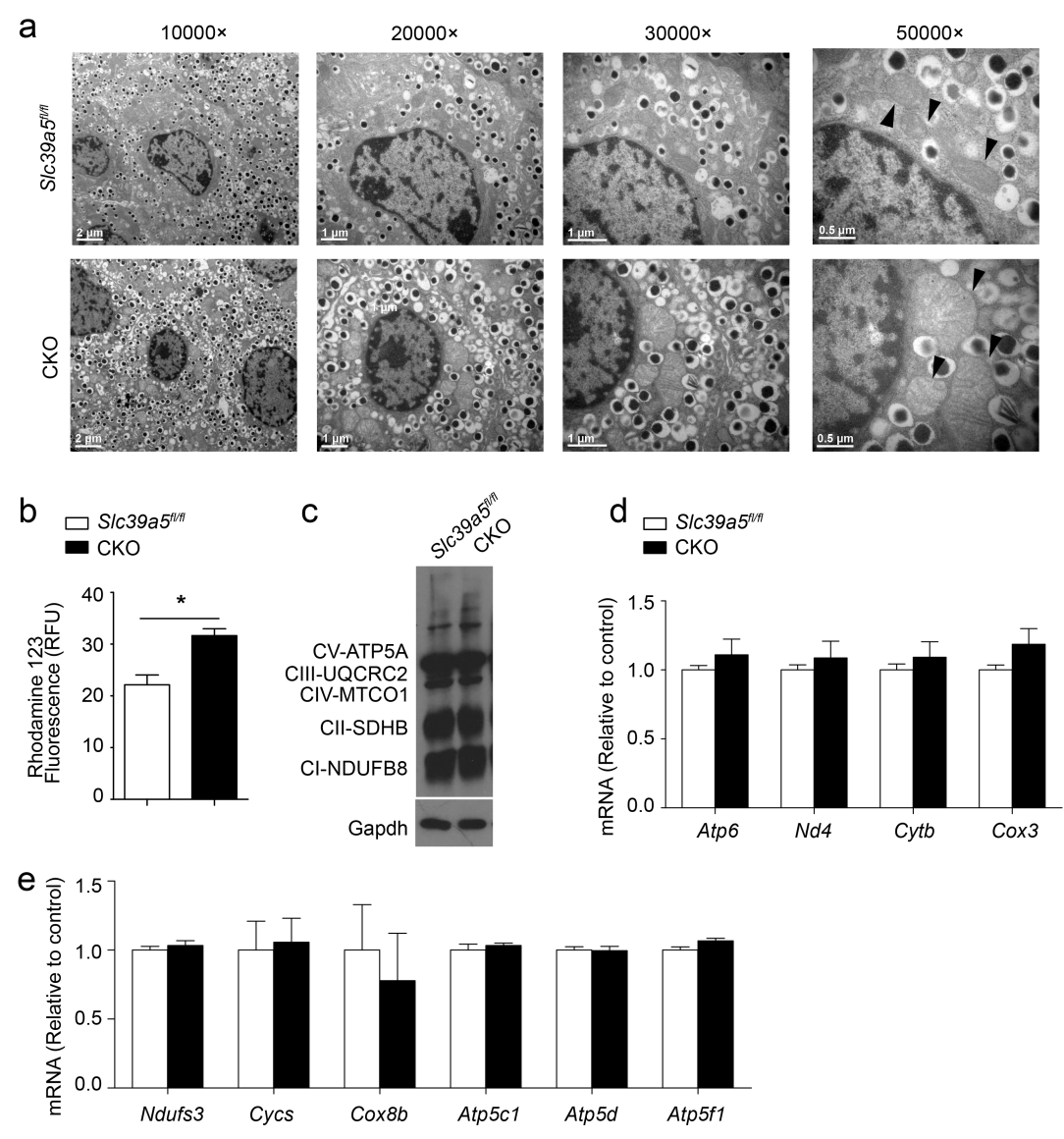


**Supplementary Figure 6. *Slc39a5*-deficient β-cells have altered mitochondrial morphology and membrane potential.** (a) Transmission electron microscopy images of mitochondria in *Slc39a5^fl/fl^* and CKO β-cells at progressively higher magnification. (b) Mitochondrial membrane potential in *Slc39a5^fl/fl^* and CKO β-cells was measured using a Rhodamine 123 uptake assay (n=4 mice per group). (c) Western blot analysis of mitochondrial complex I, II, III, IV, and V (CI through CV, respectively) proteins measured in pancreatic islets from *Slc39a5^fl/fl^* and CKO mice. (d and e) The mRNA levels of the indicated mitochondrial genes (d) and electron transfer chain complex genes (e) were measured in pancreatic islets from *Slc39a5^fl/fl^* and CKO mice (n=4 mice per group). **p* < 0.05 (Student’s *t*-test).

**Supplementary Table 1. List of primers used for quantitative RT-PCR analysis.**

| **Gene name** | **Forward primer** | **Reverse primer** |
| --- | --- | --- |
| *Hprt* | TTTCCCTGGTTAAGCAGTA | TGGCCTGTATCCAACACTTCGAGA |
| *Slc39a1* | ACTACCTGGCTGCCATAGAT | GAAACCCATGGCCAAGATGA |
| *Slc39a2* | CTGGAGGGAATTGAGTCAGAAA | AAGCAGCATCACGAGAAGAA |
| *Slc39a3* | CCTGCAGTGAGGGACAAG | GGTAGTCGGTGCTGATGTG |
| *Slc39a4* | GGACCAGCTCAGTCAAACA | GACCGAACACAGCACAGA |
| *Slc39a5* | CACCCACAAGTGGAGACAAT | CAGGTCAGGAGCCTTTGATT |
| *Slc39a6* | CGTACTCACACTGATCAAGCA | TGCTTCTTGCTCTCCACATC |
| *Slc39a7* | GACATGGACACTCCCACAG | GCGACAATCCCACTGAGAA |
| *Slc39a8* | TCTAAGAAAGCACAACGCAAAG | AGGAGAGAGGCCAGATTGATA |
| *Slc39a9* | TCATTCCTTTGGCTGTTAATTTCTC | GCAGTTCCACAGAGAAGACC |
| *Slc39a10* | ACTCTGGTTCCTGAAGATAAGAC | GCAGACTAATGACGGTGATAGA |
| *Slc39a11* | CGGAGAGTGAACTTTCCATCC | CAGTAGCTGCCACCTTCTTC |
| *Slc39a12* | AGTACTTTGGCACTTCCAGTAG | CAGATTCCCTCTGCAGAATCTTA |
| *Slc39a13* | GAAGATGTTCCTCAACAGCAAG | CAGACAGTGGCCTCCATT |
| *Slc39a14* | TTTCCCAGCCCAAGGAAG | CAAAGAGGTCTCCAGAGCTAAA |
| *Ins1* | CTTCTTCTACACACCCAAGTCC | CAGCTCCAGTTGTTCCACTT |
| *Ins2* | GAAGTGGAGGACCCACAAG | GTCTGAAGGTCACCTGCTC |
| *Gck* | ACCGGATGGTGGATGAGA | CAGCTCGCCCATGTACTTT |
| *Ucp2* | GGTGACCTATGACCTCATCAAA | CCGAAGGCAGAAGTGAAGT |
| *Sur1* | GCTGAAGCTGGTGGTGAA | CTGCCTCTGACCTTGACTAAA |
| *Kir6.2* | CTTGGAAGGCGTGGTAGAAA | TAGAATCTCGTCAGCTAGGTAGG |
| *Glut1* | TCTTAAGTGCGTCAGGGCGT | GTCACCTTCTTGCTGCTGGGAT |
| *Glut2* | TCATTGCTGGACGAAGTGTATC | ACATTGGAACCAGTCCTGAAA |
| *Glut3* | TCTGTAGGACCCGAGGAACA | GAGATGGGGTCACCTTCGTT |
| *Glut4* | GATGGGGAACCCCCTCGG | TTCAATCACCTTCTGTGGGGC |
| *Glut5* | ACAGTAAAACAGGCAGAGAGAAGA | CACGGTGGCTCTTCAATTCG |
| *Glut6* | GGGTATGCCTTGGTCTACACAT | CGGCACCCAAGGTGAACA |
| *Glut7* | TATGGCTACAACATCGCCGT | CCATCTGTTGCTTGTGCCC |
| *Glut8* | TCTTCATTGCTGGCTTTGCG | TTGGTGAGGACACAGATGCC |
| *Glut9* | AGATGCCCTGGCAAGTCC | AGGACCATTTCTTTGTCCTCCT |
| *Glut10* | TACTCGCTAGCTATGGGCCT | GGAGGGCACCAGATATGACG |
| *Glut12* | TGGACTGAGCCGAACTGAAC | CCAAGGCATGGGTCCCAGA |
| *Glut13* | CACCGCAGCTGGACCTATAA | CCCGAGAGTTGCTGGAACAT |
| *Creb1* | CCACTGATGGACAGCAGATT | CTGTGCGGATCTGGTATGTT |
| *Sirt1* | GTGAGACCAGTAGCACTAATTCC | TGAGGCAAAGGTTCCCTATTT |
| *Ppargc1a* | GAGCGAACCTTAAGTGTGGAA | AGGAGTTGTGGGAGGAGTTA |
| *Pparg* | CTGTCATTATTCTCAGTGGAGACC | CAGCAGGTTGTCTTGGATGT |
| *Nfe2l1* | CAGCAGTGGCAAGATCTCAT | GGGCATTGTACAGAATCTCACT |
| *Nfe2l2* | GCCTTGTACTTTGAAGACTGTATG | CAGGGCAAGCGACTCAT |
| *Mtf1* | AGCAAGCATCGAAAGTGGA | TGAGGCCAATCTGCTGAAC |
| *Gr* | GCAGTGAAATGGGCAAAGGCGATA | CCAGGGCAAATGCCATGAGAAACA |
| *Nr2f1* | TTCAGGAACAGGTGGAGAAGCTCA | TTTCTCCTGCAGGCTTTCGATGTG |
| *Nr2f2* | TCCAAGAGCAAGTGGAGAAGCTCA | ACTCTTCCAAAGCACACTGGGACT |
| *Pxr* | TGATGGACGCTCAGATGCAAACCT | AGAAACTCTGGAAGCTCACAGCCA |
| *Rxra* | TGACATGCAGATGGACAAGACGGA | TGCAGTACGCTTCTAGTGACGCAT |
| *Nfatc4* | ATGGTGGCTACAGCCAGCTATGAA | TCACCCTTCCGTAGCTCAATGTCT |
| *Crebbp* | ATGCCCAATGTTTCCAACGACCTG | GCCCAGCATGCAGATGAATCACAA |
| *Ppp3ca* | TGTGTACACGGTGGTTTGTCTCCA | ACAGCCTCTGACTGTGTTGTGAGT |
| *E12* | TTCCTTTGACCCTAGCCGGACATA | AACACTGGTGTCTCTCCCAAAGGT |
| *Gsk3b* | CACCTGCACTCTTCAACTTTAC | GATAAGGATGGTGGCCAGAG |
| *Ctnnb1* | CTCTTCAGGACAGAGCCAATG | CACCAATGTCCAGTCCAAGAT |
| *Tcf712* | CGTCACACCGACAGTCAAG | TTGGAGTCCTGATGCTTTGAG |
| *Tcf7* | AAGAAGAAGAGGCGGTCAAG | TCTCCGGGTAAGTACCGAAT |
| *Atp6* | AATTACAGGCTTCCGACACAAAC | TGGAATTAGTGAAATTGGAGTTCCT |
| *Nd4* | CATCACTCCTATTCTGCCTAGCAA | TCCTCGGGCCATGATTATAGTAC |
| *Cytb* | GCCACCTTGACCCGATTCT | TTGCTAGGGCCGCGATAAT |
| *Cox3* | CGGAAGTATTTTTCTTTGCAGGAT | CAGCAGCCTCCTAGATCATGTG |
| *Ndufs3* | GCTTCGAGGGACATCCTTTC | AGTTACTTGGTTTCAGGCTTCT |
| *Cycs* | AACCCATGAAGTACATGTGG | TGTAACGGAAGACAGATGGT |
| *Cox8b* | AGTTCACAGTGGTTCCCAAAG | ACCATGAAGCCAACGACTATG |
| *Atp5c1* | CATGGACAACGCCAGCAAGA | TTTACCTCTTGTCTGAGGATGCAAC |
| *Atp5d* | GCTGAAGAAGCTGTGACACT | TTGGCCTCAATACGGATCTG |
| *Atp5f1* | GTCGCAAGGAGGAAGAACAC | TCACATAATTGGCTGAGCTTGA |
